# Apparent diffusion coefficient histogram analysis for differentiating solid ovarian tumors

**DOI:** 10.3389/fonc.2022.904323

**Published:** 2022-08-01

**Authors:** Renwei Liu, Ruifeng Li, Jinzhi Fang, Kan Deng, Cuimei Chen, Jianhua Li, Zhiqing Wu, Xiaoxu Zeng

**Affiliations:** ^1^ Department of Radiology, Affiliated Longhua People’s Hospital Southern Medical University (Longhua People’s Hospital), Shenzhen, China; ^2^ C&TS Clinical Science, Philips Healthcare, Guangzhou, China

**Keywords:** diffusion weighted image (DWI), apparent diffusion coefficient (ADC), Histogram analysis, magnetic resonance imaging (MRI), solid ovarian tumors

## Abstract

**Objective:**

To evaluate the utility of apparent diffusion coefficient (ADC) histogram analysis to differentiate between three types of solid ovarian tumors: granulosa cell tumors (GCTs) of the ovary, ovarian fibromas, and high-grade serous ovarian carcinomas (HGSOCs).

**Methods:**

The medical records of 11 patients with GCTs of the ovary (regions of interest [ROI-cs], 137), 61 patients with ovarian fibromas (ROI-cs, 161), and 14 patients with HGSOCs (ROI-cs, 113) confirmed at surgery and histology who underwent diffusion-weighted imaging were retrospectively reviewed. Histogram parameters of ADC maps (ADCmean, ADCmax, ADCmin) were estimated and compared using the Kruskal-WallisH test and Mann-Whitney U test. The area under the curve of receiver operating characteristic curves was used to assess the diagnostic performance of ADC parameters for solid ovarian tumors.

**Results:**

There were significant differences in ADCmean, ADCmax and ADCmin values between GCTs of the ovary, ovarian fibromas, and HGSOCs. The cutoff ADCmean value for differentiating a GCT of the ovary from an ovarian fibroma was 0.95×10^-3^ mm^2^/s, for differentiating a GCT of the ovary from an HGSOC was 0.69×10^-3^ mm^2^/s, and for differentiating an ovarian fibroma from an HGSOC was 1.24×10^-3^ mm^2^/s.

**Conclusion:**

ADCmean derived from ADC histogram analysis provided quantitative information that allowed accurate differentiation of GCTs of the ovary, ovarian fibromas, and HGSOCs before surgery.

## Introduction

Ovarian cancer is a common cause of cancer-related mortality among women (1). Initiatives around symptom awareness and early diagnosis of ovarian cancer are critical for treatment success. Magnetic resonance imaging (MRI) has excellent soft tissue contrast and multi-directional and multi-parameter imaging (2). Conventional MRI sequences, including T1-weighted images (T1WI), T2-weighted images (T2WI), fat-suppressed T2WI and contrast-enhanced MRI, are important for the detection and characterization of ovarian tumors. In particular, conventional MRI displays morphological features that can facilitate the detection of malignant ovarian tumors, especially malignant epithelial tumors (3–5). However, morphological features on conventional MRI lack specificity and do not represent quantitative objective measures (6).

Diffusion-weighted MRI (DWI) has important clinical applications. DWI is a non-invasive MRI method that can measure the diffusion of water molecules across tissues, *in vivo*. The motion of water molecules in tissues depends on tissue cellularity and the integrity of cell membranes. The differences in the motion of water molecules between tissues determine DWI signal attenuation.

DWI displays microscopic changes in tumor biology at the cellular and molecular levels, which may aid in preoperative diagnosis (7–9). DWI has applications in the abdomen (10), including for the differentiation of benign and malignant ovarian tumors and the staging of malignant tumors (11). For solid ovarian tumors or cystic solid tumors, DWI provides qualitative data on the structural components, and apparent diffusion coefficient (ADC) values provide quantitative data on histological characteristics. However, the extent to which ADC values can be used to discern benign and malignant tumors remains controversial (11). In one study that used diffusion-weighted echoplanar imaging to evaluate cystic ovarian lesions, endometrial cysts and malignant cystic ovarian tumors showed significantly lower ADC values than ovarian cysts and serous cystadenomas (12). Other studies have shown ADC values for solid components of malignant ovarian tumors are low, with no significant difference in ADC values between benign and malignant ovarian tumors and an overlap between ADC values of borderline tumors and benign and malignant tumors (13–15).

ADC histogram analysis is useful in characterizing cancers. This approach includes an entire lesion and reflects the heterogeneity of a tumor (16–18). The objective of this study was to evaluate the utility of ADC histogram analysis to differentiate between three types of solid ovarian tumors: granulosa cell tumors (GCTs) of the ovary, ovarian fibromas, and high-grade serous ovarian carcinomas (HGSOCs).

## Materials and methods

### Study subjects

The medical records of female patients diagnosed with solid ovarian tumors between 1 January 2017 and 9 February 2022 were retrospectively reviewed. Inclusion criteria were: 1) unilateral or bilateral solid ovarian tumor; 2) no history of surgery; 3) no history of other tumors or systemic diseases (e.g., gastric cancer, colon cancer, endometrial carcinoma); and 4) no evidence of pelvic lymph node metastasis, reactive lymph node hyperplasia or pelvic lymph node enlargement due to other causes.

All patients underwent MRI examination 3-7 days prior to laparoscopy or surgery. Patients were divided into three groups based on pathological findings, Group A, GCTs of the ovary (adult type); Group B: ovarian fibromas; and Group C: HGSOCs.

The protocol for this study was reviewed and approved by our institutional review board.

Patients were scanned using a 3.0T (Tesla) superconducting MRI scanner (Philips Ingenia). Scanning parameters are summarized in **Table 1**. T2WI-SPIR was used as the morphological sequence. DWI sequences included b-values of 0 and 800s/mm^2^. DWI parameters: patient position: feet first, patient orientation: supine, diffusion mode: DWI, fat suppression: SPAIR, fast imaging mode (SE): EPI, EPI factor: 49, shot mode: single-shot, TR (ms): 5500, min TR (ms): 5337, TE (ms): 66, flip angle (deg): 90, NSA: 2, SNR: 1, FOV (mm): 230×230×123 (RL×AP×FH), voxel size (mm): 2.5×2.09×3.5 (RL×AP×FH), recon voxel size RL\AP: 0.8\0.8, REC voxel MPS (mm): 0.80/0.80/3.50, ACQ voxel MPS (mm): 2.50/2.14/3.50, matrix (slices): 216×128×32 (RL×AP×FH), reconstruction matrix: 288, ACQ matrix M×P: 92×107, slice thickness (mm): 3.5, slice orientation: transverse, fold-over direction: AP, fat shift direction: A, min slice gap (mm): 0, ACT slice gap (mm): 0.35, scan percentage (%): 116.7, total scan duration: 01:22.5, WFS (pix)/BW(Hz): 14.335/30.3, BW in EPI freq.dir (Hz): 3153.6, SPAIR offset act/default: 250[220], local torso SAR: <79%, whole body SAR/level: <2.1W/kg/1 st level, SED: <0.2kj/kg, coil power: 63%, max B1+rms: 1.85uT, PNS/level: 77%/normal, Db/dt: 53.4T/s, sound pressure level (dB): 18.2. ADC maps were processed using post-processing software (Philips Intellispace Portal). Two radiologists placed a region of interest (circular ROI [ROI-c]) (10mm^2^-300mm^2^) on the ADC map of each ovarian solid tumor. A histogram was made for each ROI. The area of the ROI (mm^2^), ADCmean, ADCmax, ADCmin and standard deviation (SD) were calculated.

**Table 1:** Scanning parameters.

SEQUENCE	mDIXON-T1WI	TSE-T2WI	TSE-T2WI-SPIR	TSE-T2WI-SPIR	DWI (b0/800s/mm^2^)	mDIXON-T1WI+CE
Plane	TRA	TRA/SAG	TRA	COR	TRA	TRA
TR (ms)	SHORTEST	SHORTEST	SHORTEST	SHORTEST	5500	SHORTEST
TE (ms)	SHORTEST	70~90	SHORTEST	SHORTEST	SHORTEST	SHORTEST
ST (mm)	4	4	3.5	3	3.5/4	4
Gap (mm)	0	0.4	0.35	0.3	0.35	0
Matrix (mm)	240~250× 150~160	180~200× 150~170	160~180× 160~180	220~240× 220~240	80~100× 100~110	240~250× 150~160
FOV (mm)	300×250	250×250	230×230	300×300	230×230	300×250

CE, Contrast enhancement; Gd-DTPA, Gadolinium-diethylenetriaminepentaacetate 0.2 mmol/kg, flow rate 2.0mL/s; TR, repetition time; TE, echo time; ST, Slice thickness; FOV, field of view; TRA, Transverse; COR, Coronary; SAG, Sagittal.

### Statistical analysis

Statistical analysis was conducted using SPSS v28.0.1. The normality of the ADC histogram parameters was evaluated with the single-sample Shapiro-Wilk test. Normally distributed data with homogeneity of variance were compared with ANOVA. Non-normally distributed data with heterogeneous variances were compared with the non-parametric Kruskal-WallisH test. Pairwise comparison was made with the Mann-Whitney U test. The area under the curve (AUC) of receiver operating characteristic (ROC) curves was used to assess the diagnostic performance of ADC parameters for solid ovarian tumors. *P*<0.05 was considered statistically significant.

## Results

Medical records of 86 patients with solid ovarian tumors were reviewed, including 11 patients with GCTs of the ovary, 61 patients with ovarian fibromas, and 14 patients with HGSOCs. A total of 411 ROI-cs were evaluated, including 137 GCT of the ovary ROI-cs, 161 ovarian fibroma ROI-cs, and 113 HGSOC ROI-cs. Patients mean (SD) age was 45.47 ± 3.45 years (range, 22-64 years old), and time since diagnosis ranged from 3 weeks to 2 months. 71 patients underwent open abdominal surgery, and 15 patients underwent laparoscopic surgery.

**Figure 1:**
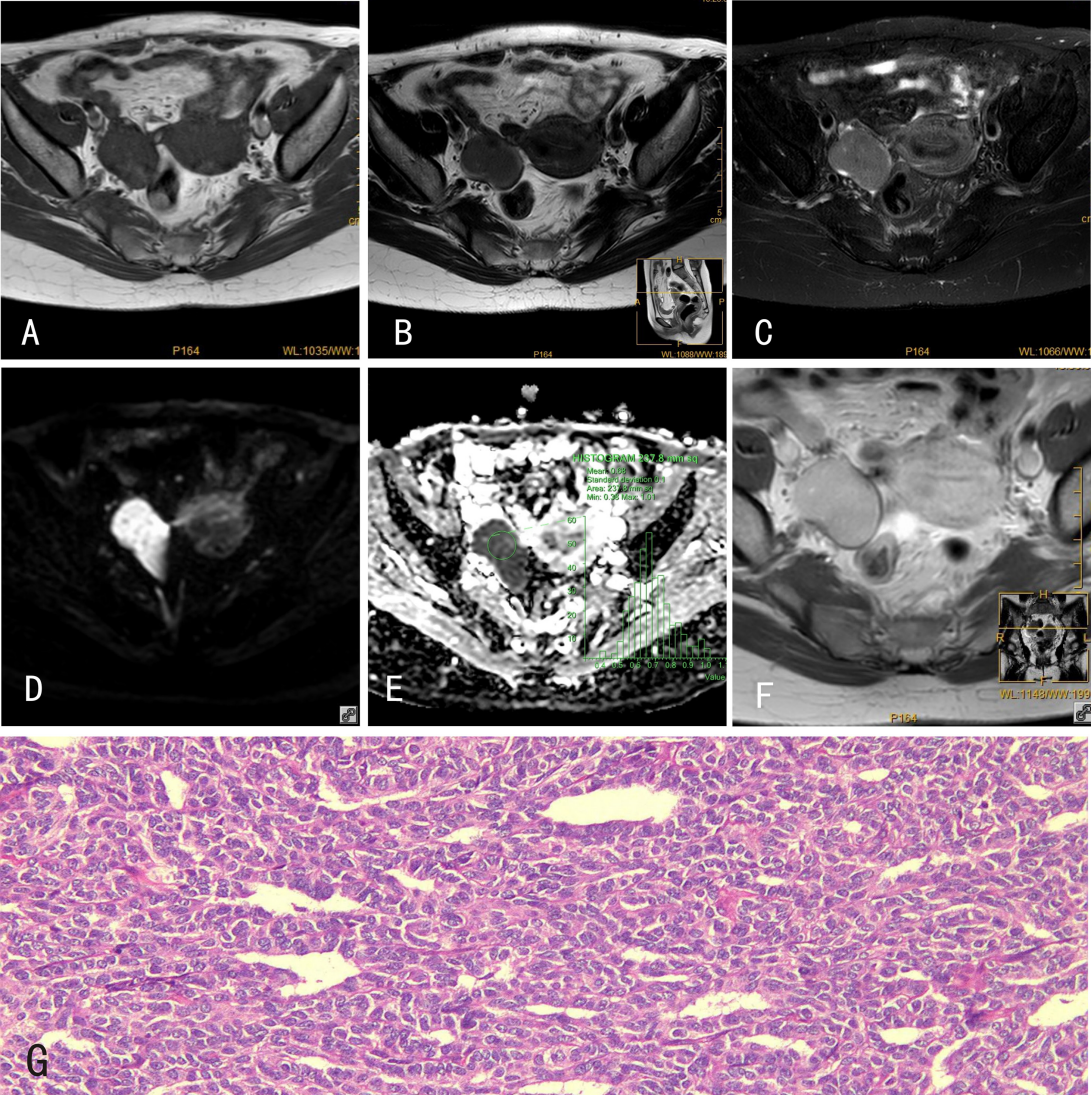
GCTs of the ovary. 34-year-old female with a GCT of the right ovary. **(A)** The GCT showed an isointense signal on mDIXON-T1WI; **(B)** slightly hyperintense signal on TSE-T2WI; **(C)** hyperintense signal on TSE-T2WI-SPIR; **(D)** hyperintense signal on DWI (b=800s/mm^2^); and **(E)** hypointense signal on the ADC map. **(F)** The GCT was markedly enhanced after Gd-DTPA administration. **(G)** Histological features of GCTs of the ovary, HE×100, tumor cells are tightly packed and have a”coffee bean”-like nucleus.

**Figure 2:**
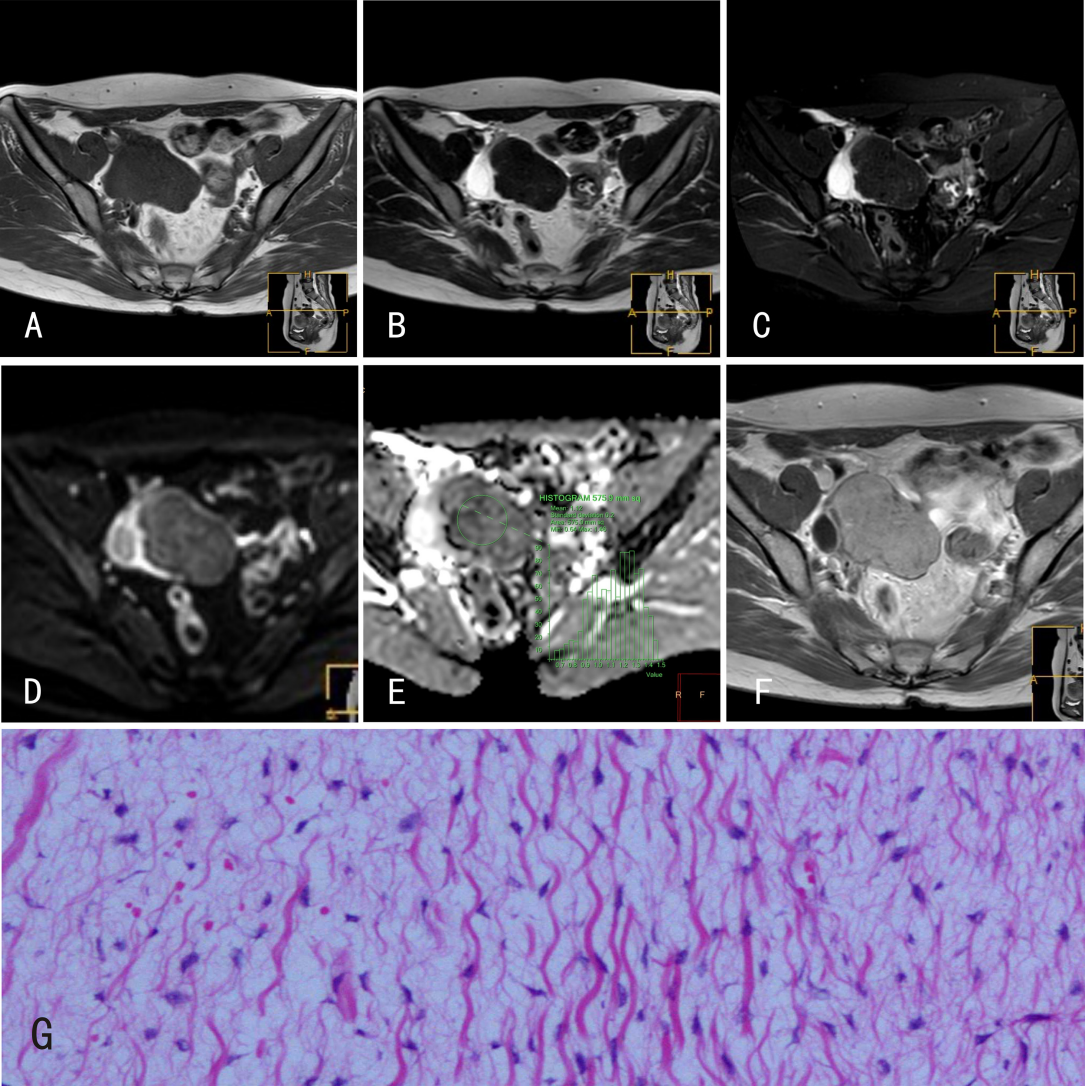
Ovarian fibromas. 22-year-old female with a right ovarian fibroma. **(A)** The ovarian fibroma showed an isointense signal on mDIXON-T1WI; **(B)** isointense signal on TSE-T2WI; **(C)** isointense signal on TSE-T2WI-SPIR; **(D)** hyperintense signal on DWI (b=800s/mm^2^); and **(E)** isointense signal on the ADC map. **(F)** The ovarian fibroma was markedly enhanced after Gd-DTPA administration. **(G)** Histological features of ovarian fibromas, HE×100, tumor cells are spindle shaped and loosely arranged such that they appear as “tadpoles” surrounded by significant interstitial edema.

**Figure 3:**
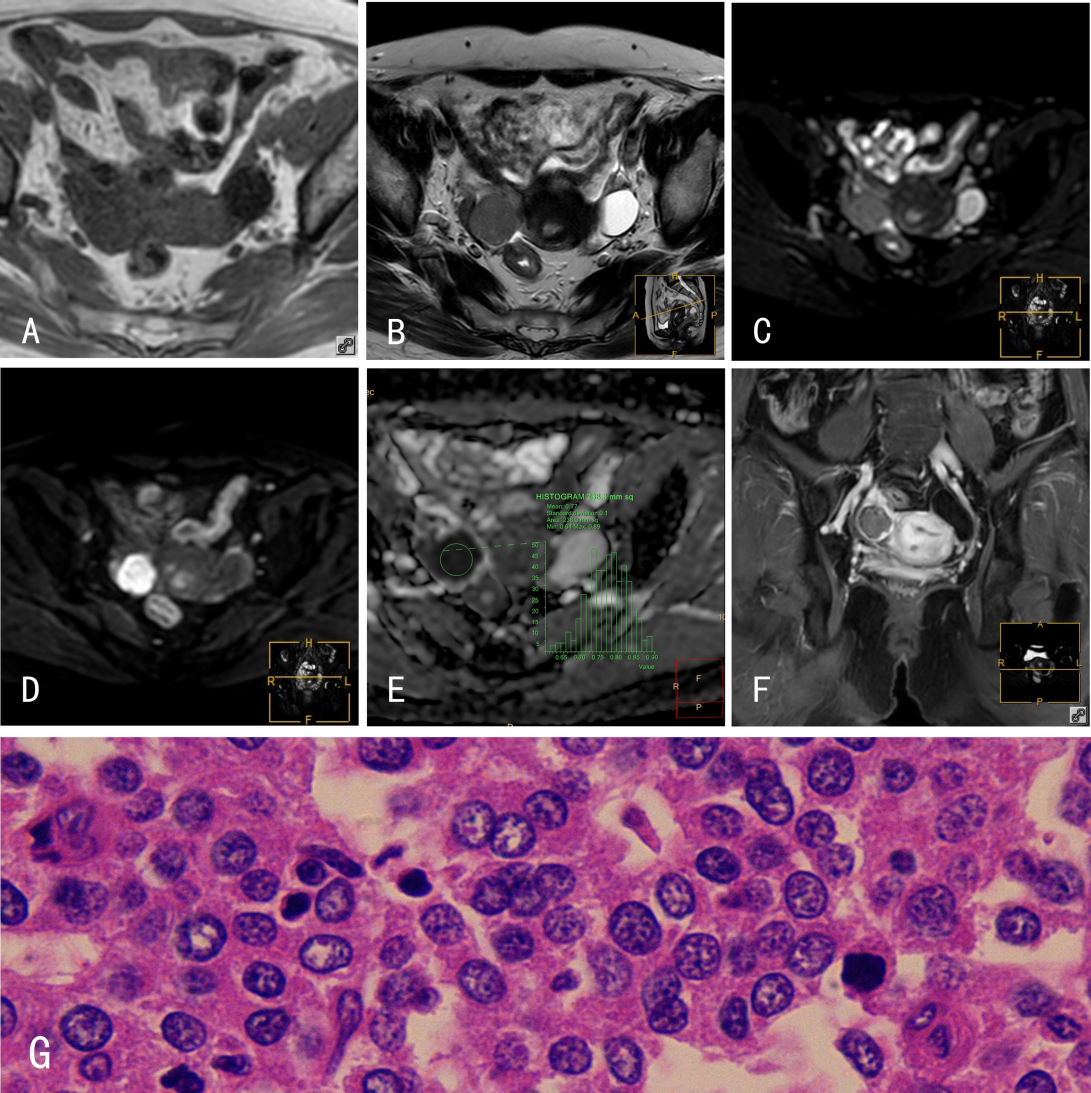
HGSOCs. 45-year-old female with a right HGSOC. **(A)** The HGSOC showed an isointense signal on mDIXON-T1WI; **(B)** slightly hyperintense signal on TSE-T2WI; **(C)** slightly hyperintense signal on TSE-T2WI-SPIR; **(D)** hyperintense signal on DWI (b=800s/mm^2^); and **(E)** hypointense signal on the ADC map. **(F)** The HGSOC was slightly enhanced after Gd-DTPA administration. **(G)** Histological features of HGSOCs, HE×400; tumor cells are loosely arranged with some in mitosis. The glands are disordered with branching papillary structures and stromal infiltrates.

Pathological findings revealed 84 patients had unilateral solid ovarian tumors and 2 patients had bilateral ovarian tumors. Histological features of the solid ovarian tumors are shown in **Figures 1**–**3**.

Results of the tests for normality and variance homogeneity are shown in **Supplementary Tables 1**, **2**. ADCmin for ovarian fibromas and HGSOCs were normally distributed **(** **Supplementary Table 1** **)** but did not conform to assumptions of homogeneity of variance (**Supplementary Table 2**).

Comparison of ADC histogram parameters among GCTs of the ovary, ovarian fibromas, and HGSOCs are shown in **Tables 2**, **3**. There were no significant differences in the area of the ROI-cs (*P*=0.052) between GCTs of the ovary, ovarian fibromas, and HGSOCs, but there were significant differences in the other ADC parameters (ADCmean, ADCmax, ADCmin, and SD). The diagnostic performance of significant ADC histogram parameters for differentiating between GCTs of the ovary, ovarian fibromas, and HGSOCs is shown in **Supplementary Table 3** (SD was excluded due to its lack of clinical utility). ROC curve analysis **(** **Supplementary Figure 1** **)** and the Youden index were used to determine optimum cutoff values.

**Table 2:** Kruskal-wallisH test.

Parameter	kruskal-wallisH test	variance	*P* value
ROI-c	6.345	2	0.052
ADCmean	314.681	2	<0.0001
ADCmax	289.862	2	<0.0001
ADCmin	292,412	2	<0.0001
SD	237,987	2	<0.0001

**Table 3:** ADC histogram parameters of three ovarian solid tumors.

GROUP	ADCmean	ADCmax	ADCmin	SD
GCT of the ovary (n=11)	0.43 (0.38~0.63)	0.77 (0.665~0.9)	0.33 (0.285~0.42)	0 (0~0.1)
Ovarian fibroma (n=61)	1.58 (1.245~1.865)	1.97 (1.605~2.44)	1.09 (0.945~1.31)	0.2 (0.1~0.3)
HGSOC (n=14)	1.08 (0.89~1.15)	1.49 (1.26~1.73)	0.78 (0.63~0.835)	0.1 (0.1~0.2)
	-14.752^a^	-14.760^a^	-14.287^a^	-13.993^a^
*Z* value	-12.732^b^	-12.465^b^	-12.272^b^	-11.838^b^
	-11.047^c^	-8.646^c^	-10.486^c^	-5.122^c^
	<0.0001^a^	<0.0001^a^	<0.0001^a^	<0.0001^a^
*P* value	<0.0001^b^	<0.0001^b^	<0.0001^b^	<0.0001^b^
	<0.0001^c^	<0.0001^c^	<0.0001^c^	<0.0001^c^

Mann- Whitney U test.

a GCT of the ovary vs. ovarian fibroma.

b GCT of the ovary vs. HGSOC.

c ovarian fibroma vs. HGSOC.

The cutoff ADCmean value for differentiating a GCT of the ovary from an ovarian fibroma was 0.95×10^-3^ mm^2^/s. The sensitivity, specificity, and Youden index for diagnosing a GCT of the ovary were 97%, 98%, and 95.2%, respectively. The cutoff ADCmean value for differentiating a GCT of the ovary from an HGSOC was 0.69×10^-3^ mm^2^/s. The sensitivity, specificity, and Youden index for diagnosing a GCT of the ovary were 88%, 100%, and 88.3%, respectively. The cutoff ADCmean value for differentiating an ovarian fibroma from an HGSOC was 1.24×10^-3^ mm^2^/s. The sensitivity, specificity, and Youden index for diagnosing ovarian fibroma were 76%, 0.91%, and 67.5%, respectively. The AUC under the ROC curve of ADCmean values for differentiating a GCT of the ovary from an ovarian fibroma was 0.996 (95% CI, 0.991-1.000). The AUC under the ROC curve of ADCmean values for differentiating a GCT of the ovary from an HGSOC was 0.968 (95% CI, 0.947-0.988). The AUC under the ROC curve of ADCmean values for differentiating an ovarian fibroma from an HGSOC was 0.892 (95% CI, 0.855-0.929). The AUC under the ROC curve of ADCmax values for differentiating a GCT of the ovary from an ovarian fibroma was 0.996 (95% CI, 0.992-1.000).

## Discussion

Ovarian solid tumors have variable histological types, and include benign and malignant tumors (19). Ovarian solid tumors may have similar appearances on MRI, and accurate diagnosis may be difficult.

In the present study, ADC histogram analysis provided quantitative information that allowed accurate differentiation of GCTs of the ovary, ovarian fibromas and HGSOCs before surgery, with ADCmean having the highest value for discriminating between the three types of solid ovarian tumors. ADCmax also proved useful for discriminating between GCTs of the ovary and ovarian fibromas.

GCTs of the ovary are low-grade malignant ovarian sex cord stromal tumors. An estimated 64%-89% of GCTs of the ovary are Federation International of Gynecology and Obstetrics Stage I (20). Surgery is the primary treatment for GCTs of the ovary, with the goal of removing the primary tumor and metastases. Most recurrences occur within 5-10 years of initial treatment (20). Ovarian fibromas are benign pure stromal tumors. Choice of treatment is determined by the woman’s desire to preserve future fertility. Ovarian fibromas are completely curable *via* surgery (21). HGSOCs are aggressive malignant ovarian tumors. Standard therapy includes cytoreductive surgery and platinum-based chemotherapy (22, 23).

Preoperative MRI is used to determine the anatomic location and size of ovarian tumors and the extent of tumor dissemination within the pelvis. Routine preoperative pelvic MR scanning sequences include T1WI, T2WI, T2WI-SPAIR, and T1WI+ contrast enhancement (CE). These sequences provide high spatial resolution and soft tissue discrimination but cannot differentiate pathological types and benign and malignant tumors. T1WI and T2WI are useful for detecting chocolate cysts and mature teratomas of the ovary, but may not fully reflect the characteristics of the tissue inside an ovarian tumor. T2WI-SPAIR sequences increase contrast, which can highlight the morphology and size of an ovarian tumor and the anatomic relationship between the tumor and the ovary/uterus. T1WI+CE can be used to evaluate tumor blood flow and display cystic components and areas of necrosis, which inform clinical decision making; however, it is of limited value for pathological diagnoses. Manifestations of GCTs of the ovary, ovarian fibromas and HGSOCs overlap on conventional MRI sequences, and there is an unmet clinical need to develop MRI sequences that provide accurate quantitative evaluation of solid ovarian tumors. ADC values may have utility for differentiating ovarian granulosa cell tumors (OGCT) from other ovarian sex cord-stromal tumors (24).

Recent evidence implies ADC histogram analysis has clinical utility in the preoperative classification of solid ovarian tumors, prediction of lymph node metastasis, and assessment of chemotherapy response (25). Consistent with the findings reported here, previous studies showed ADC histogram parameters differed between stage I and stage II, III and IV epithelial ovarian cancer, were significantly lower in lymph node-positive compared to lymph node-negative patients, were significantly negatively correlated with the Ki-67 labeling index, and were significantly lower in patients with mutated p53 compared to wild-type p53 (25); ADC histogram parameters based on whole solid tumor volume may have utility for differentiating between HGSOC and low-grade serous ovarian carcinoma (LGSOC) (26); and in patients with advanced HGSOC, pretreatment ADC histogram analysis of primary tumors had potential for predicting response to platinum-based chemotherapy (27).

The ADC histogram parameters reported here align with those described in previous studies. Specifically, the ADCmean for HGSOC was consistent with the ADCmean for Stage III-IV epithelial ovarian cancer (25) and another patient population with HGSOC (26), ADCmax was consistent with the ADCmax for Stage I-II epithelial ovarian cancer (25), and the ADCmax for GCT of the ovary was consistent with the ADC value for OGCT (GCT of the ovary) on DWI using an echo-planar imaging two-dimensional (EP2D) sequence performed in the axial plane with parallel acquisition technique and b values of 0, 100, and 800 s/mm^2^ (0.817 ± 0.144 [0.558-1.120]) (24).

The motion of water molecules in tissues depends on tissue cellularity and the integrity of cell membranes (28–30). Consequently, ovarian tumor cellularity should correlate with ADC values. Accordingly, in the present study, ADC values reflected pathology images, which showed GCTs of the ovary had high cell densities and relatively large nuclei, while ovarian fibroma and HGSOC tumor cells were more dispersed with obvious interstitial edema. The correlation of DWI parameters with markers of proliferation (Ki67) and factors influencing angiogenesis such as VEGF within tumors, as well as the significant correlation of ADC values with serous epithelial ovarian cancer (EOC) type (low-grade vs. high-grade), make MRI an excellent tool in the diagnosis of serous ovarian cancer (31).

This study was associated with several limitations. First, it was a retrospective study, and the clinical value of ADC histogram analysis for discriminating between solid ovarian tumors should be verified prospectively. Second, the sample size was small and there may have been interobserver variability with regard to ROI-cs selection, which may have introduced bias. Third, DWI sequences included b-values of 0 and 800s/mm^2^; further research should include multi-b-values DWI. Fourth, distortion and deformation often occur at high-b-values, which may have influenced ADC histogram parameters Last, this study only used three ADC histogram parameters (ADCmean, ADCmin and ADCmax), without further discussion of skewness and kurtosis.

In conclusion, ADCmean derived from ADC histogram analysis provided quantitative information that allowed accurate differentiation of GCTs of the ovary, ovarian fibromas, and HGSOCs before surgery.

## Data availability statement

The raw data supporting the conclusions of this article will be made available by the authors, without undue reservation.

## Ethics statement

The studies involving human participants were reviewed and approved by the institutional review board of People’s Hospital of Longhua (Ky2022307). The patients/participants provided their written informed consent to participate in this study.

## Author contributions

RWL: Statistics, manuscript writing; RFL: Literature retrieval; JF: MR scanning; KD: Software Technical Support; CC: MR scanning; JL: MR scanning; ZW: MR scanning; XZ: The paper design; All authors contributed to manuscript revision, read, and approved the submitted version.

## Conflict of interest

The authors declare that the research was conducted in the absence of any commercial or financial relationships that could be construed as a potential conflict of interest.

## Publisher’s note

All claims expressed in this article are solely those of the authors and do not necessarily represent those of their affiliated organizations, or those of the publisher, the editors and the reviewers. Any product that may be evaluated in this article, or claim that may be made by its manufacturer, is not guaranteed or endorsed by the publisher.

## Supplementary material

Supplementary Figure 1 ROC curve analysis. ^1^Differentiating between GCT of the ovary and. ovarian fibroma ^2^ Differentiating between GCT of the ovary and HGSOC ^3^Differentiating between ovarian fibroma and HGSOC.
