# Supplementary material for: Apparent diffusion coefficient histogram analysis for differentiating solid ovarian tumors
Source: Front Oncol. 2022 Aug 1;12:904323. doi: 10.3389/fonc.2022.904323 (PMC9376384; doi:10.3389/fonc.2022.904323)
Supplement: Supplementary file 1 [file Table_1.docx]

| parameter | Group | ShapIro-wilk | | |
| --- | --- | --- | --- | --- |
|  |  | Statistics | Variance | *P* value |
|  | GCT | 0.887 | 137 | <0.0001 |
| ROI-c | ovarian fibroma | 0.655 | 161 | <0.0001 |
|  | HGSOC | 0.799 | 113 | <0.0001 |
|  | GCT | 0.865 | 137 | <0.0001 |
| ADCmean | ovarian fibroma | 0.963 | 161 | <0.0001 |
|  | HGSOC | 0.955 | 113 | 0.001 |
|  | GCT | 0.906 | 137 | <0.0001 |
| ADCmax | ovarian fibroma | 0.966 | 161 | 0.001 |
|  | HGSOC | 0.972 | 113 | 0.019 |
|  | GCT | 0.868 | 137 | <0.0001 |
| ADCmin | ovarian fibroma | 0.984 | 161 | **0.068** |
|  | HGSOC | 0.978 | 131 | **0.065** |
|  | GCT | 0.592 | 137 | <0.0001 |
| SD | ovarian fibroma | 0.833 | 161 | <0.0001 |
|  | HGSOC | 0.790 | 113 | <0.0001 |

**Supplementary Table 1** Normality test

**Supplementary Table 2** Homogeneity of variance test: ADCmin for ovarian fibromas and HGSOCs

| Parameter | ADCmin | | | |
| --- | --- | --- | --- | --- |
|  | Based on average | Based on median | Based on median and adjusted variance | Based on post-cut average |
| Levin statistics | 32.57 | 32.299 | 32.299 | 32.770 |
| Variance 1 | 1 | 1 | 1 | 1 |
| Variance 2 | 272 | 272 | 219.188 | 272 |
| *P* value | <0.0001 | <0.0001 | <0.0001 | <0.0001 |

| **Supplementary Table 3** Diagnostic performance of ADC histogram parameters for differentiating between GCTs of the ovary, ovarian fibromas, and HGSOCs | | | | | | | | |
| --- | --- | --- | --- | --- | --- | --- | --- | --- |
| ADC-value | Comparison group | AUC | Cutoff | Sensitivity | Specificity | Youden index | 95% CI | *P* value |
| ADCmean | a | 0.996 | 0.95 | 0.97 | 0.98 | 0.952 | 0.991~1 | <0.0001 |
|  | b | 0.968 | 0.69 | 0.88 | 1.00 | 0.883 | 0.947~0.988 | <0.0001 |
|  | c | 0.892 | 1.24 | 0.76 | 0.91 | 0.675 | 0.855~0.929 | <0.0001 |
| ADCmin | a | 0.980 | 0.63 | 0.93 | 0.98 | 0.902 | 0.962~0.998 | <0.0001 |
|  | b | 0.951 | 0.47 | 0.85 | 0.96 | 0.811 | 0.924~0.977 | <0.0001 |
|  | c | 0.872 | 0.94 | 0.77 | 0.94 | 0.708 | 0.830~0.914 | <0.0001 |
| ADCmax | a | 0.996 | 1.36 | 0.98 | 0.96 | 0.941 | 0.992~1 | <0.0001 |
|  | b | 0.958 | 1.05 | 0.91 | 0.87 | 0.780 | 0.938~0.979 | <0.0001 |
|  | c | 0.807 | 1.78 | 0.67 | 0.84 | 0.512 | 0.757~0.857 | <0.0001 |
| **Note**:  ^a^ Differentiating between GCT of the ovary and. ovarian fibroma  ^b^ Differentiating between GCT of the ovary and HGSOC  ^c^ Differentiating between ovarian fibroma and HGSOC  95% CI: confidence interval | | | | | | | |  |
