# Supplementary figures and images for: Apparent diffusion coefficient histogram analysis for differentiating solid ovarian tumors
Source: Front Oncol. 2022 Aug 1;12:904323. doi: 10.3389/fonc.2022.904323 (PMC9376384; doi:10.3389/fonc.2022.904323)

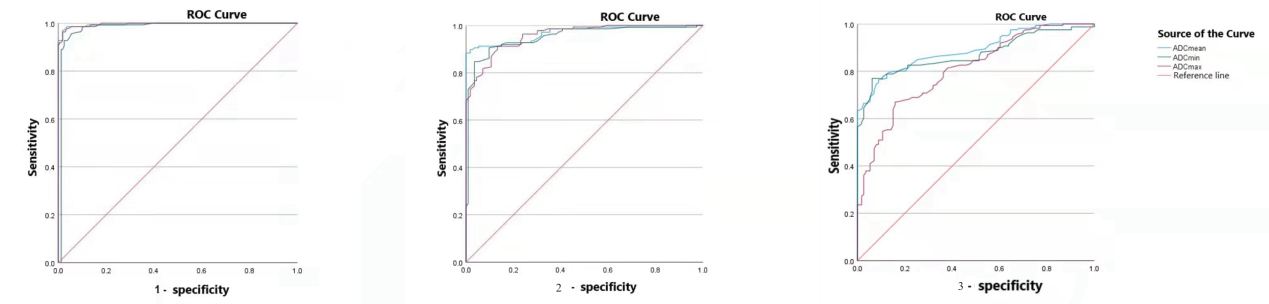

Supplement: Supplementary Figure 1 — ROC curve analysis. 1Differentiating between GCT of the ovary and. ovarian fibroma 2 Differentiating between GCT of the ovary and HGSOC 3Differentiating between ovarian fibroma and HGSOC. [file Image_1.tif]
